# Respiratory tract infection during Hajj

**DOI:** 10.4103/1817-1737.49412

**Published:** 2009

**Authors:** Abdulaziz H. Alzeer

**Affiliations:** *Department of Critical Care, KKUH, King Saud University, P.O. Box 18321, Riyadh 11415, Saudi Arabia*

**Keywords:** Hajj, respiratory tract infection, influenza, pilgrims

## Abstract

Respiratory tract infection during Hajj (pilgrimage to Mecca) is a common illness, and it is responsible for most of the hospital admissions. Influenza virus is the leading cause of upper respiratory tract infection during Hajj, and pneumonia can be serious. Taking into account the close contacts among the pilgrims, as well as the crowding, the potential for transmission of *M. tuberculosis* is expected to be high. These pilgrims can be a source for spreading infection on their return home. Although vaccination program for influenza is implemented, its efficacy is uncertain in this religious season. Future studies should concentrate on prevention and mitigation of these infections.

Hajj is a religious duty undertaken by Muslims at least once in their lifetime, and it is one of the largest annual gatherings in the world. It is performed in the 12^th^ lunar month of each year and lasts for 5 days. Approximately 2.5 million people from different parts of the world with diverse medical and social backgrounds travel to Mecca and congregate together to perform their rituals in a small area defined for the pilgrimage. Pilgrims start Hajj by visiting the sacred Ka'aba; and then on subsequent days, they move to different holy places, including Mina, Arafat and Muzdalifa. They typically stay in tent-type housing; 50 to 100 persons share domestic facilities and move around by buses or on foot. Thus crowding, fatigue and the extreme climatic conditions are important factors for transmitting air- and droplet-borne infections. Respiratory tract infections are, in fact, one of the leading types of infections seen during the Hajj season.[1,2] This imposes considerable burden by way of increased health-care costs and hospital bed demand, as well as lost workdays, and may disseminate infection across continents. This review discusses upper and lower respiratory tract infections occurring during the Hajj season, and their effect on pilgrims' health.

## Upper Respiratory Tract Infection

Acute upper respiratory tract infection (ARI) is a common illness during Hajj.[3] There are no comprehensive studies on the epidemiology and statistics of ARI during Hajj. As most data were obtained from cross-sectional studies on a relatively small number of patients [Table 1],[3–7] it has been estimated that 1 in 3 pilgrims will experience respiratory symptoms,[5] which usually start at the end of, or shortly after the end of, the Hajj season. Typical symptoms include cough, sputum production, sore throat, hoarseness of voice, rhinorrhea, fever and malaise. Cough may persist for several weeks; and if it is accompanied by purulent sputum, it suggests the possibility of a superimposed bacterial infection. Respiratory infections could be complicated by exacerbations of asthma, chronic obstructive pulmonary disease, sinusitis and pneumonia.

**Table 1:** Viral respiratory tract infections during Hajj seasons

Authors	Hajj season	Source	No. of samples	Methods of detection	Confirmed cases	Influ A (%)	Influe B (%)	Para-infl (%)	Adeno	RSV (%)	HSV (%)	Rhino virus (%)
El Sheikh *et al.*	1991	Throat swab	761	Monoclonal antibodies		Yes			Yes			
Kholeidi *et al.*	2001	Venous blood	305	ELISA	45	12 (27)	27 (60)					
Balkhy *et al.*	2003	Throat swab	500	Viral culture	54	3 (5.6)	27 (50)	4 (7.4)		7 (13)	13 (24.1)	
AlSaleh *et al.*	2004	Throat swab	360	Viral culture	46	12 (27.3)	34 (72.7)					
Rashid *et al.*	2005	Nasal swab	202	RT-PCR	37	21 (56.7)	7 (19)			9 (24)		
Rashid *et al.*	2006	Nasal swab	260	rtRT-PCR	52	22 (42)	6 (11.5)	1 (1.9)		1 (1.9)		22 (42)

Several transmissible viral respiratory infections have been reported to cause these illnesses [Table 1]. Influenza viruses are the most common, followed by respiratory syncytial viruses (RSV) and adenoviruses.[3,8] Rhinovirus infection was more commonly reported among UK pilgrims as compared to influenza, and the attack rate of ARI was higher when compared with local Saudi pilgrims (25% *vs.* 13%, respectively).[7] The higher rate of ARI in UK pilgrims was partly attributed to the longer time spent by them in Mecca and the Grand Mosque as compared to the local pilgrims.[7] In the Hajj season of 2003, Balkhy *et al.* examined the throat swabs of 500 patients with upper respiratory tract infection and found 54 (10.8%) patients with positive cultures, including 27 (50%) influenza B, 13 (24.1%) herpes simplex virus, 7 (12.9%) RSV, 4 (7.4%) parainfluenza and 3 (5.6%) influenza A.[5] In the 2004 season, Al Saleh *et al.* isolated influenza type B in 72.7% of the 46 confirmed influenza cases; with Sichuan as the predominant serotype (70.9%), followed by Flu A (not typed, 14.6%; Flu A H1N1, 7.3%; Flu A H3N2, 5.5%) and Flu B Hong Kong (1.8%).[6] In contrast, during the 2005 season, Rashid *et al.* examined nasal swabs of 205 patients using real-time polymerase chain reaction (RT-PCR). Influenza A accounted for 56.7% (21/37) of the confirmed cases, followed by RSV (24%, 9/37) and influenza B accounted for (18.9%, 7/37).[8] The main influenza strains were different in these years, suggesting that as with typical seasonal influenza epidemics, the circulating viruses are different in various years. If these data are extrapolated to the total number of pilgrims during these years, the authors estimated that at least 400,000 pilgrims would develop ARI symptoms, and 24,000 would develop influenza in one season.[5]

The magnitude of viral illnesses occurring during Hajj has the potential for triggering an influenza pandemic. It would seem prudent to regard all Hajj pilgrims to be at risk. Therefore, collaborative measures nationally and internationally should be undertaken to prevent possible pandemics, particularly with the recent breakout of the H5N1 strain in certain countries and the potential threat of its transmission during Hajj gatherings.[9] The World Health Organization (WHO) should work closely with Saudi health authorities to minimize the spread of these viruses, as the centers for Disease Control and Prevention (CDC) does not recognize Saudi Arabia as 1 of the 112 national influenza centers.[10] It is also important to identify surveillance studies as indicators of activity for a possible influenza pandemic.

The role of the influenza vaccine has been established in reducing mortality and morbidity of influenza.[11] Both inactivated and live attenuated vaccine prevented about 70% of cases of laboratory-confirmed symptomatic influenza in healthy adults.[12] Recent data regarding UK pilgrims showed that the rate of influenza was lower in a vaccinated group as compared to an unvaccinated group (7% and 14%, respectively).[7] Rashid *et al.* studied 538 pilgrims in 2005 and 2006. They found that 5% of the vaccinated pilgrims, compared to 14% of the unvaccinated pilgrims, in the “at high risk” group (underlying immunosuppression and or age over 65 years) developed whereas in the “not at risk” group 10% of vaccinated vs. 11% of unvaccinated pilgrims devoloped influenza. But the difference was not statistically significant for both high and low risk groups.[13] Others have reached the same conclusion.[7,14] The lack of statistical significance in these studies was attributed to the mismatch between the vaccine strain(s) and the circulating strain(s), small sample size and disparities between the vaccinated (sicker) and unvaccinated (healthy) groups.[7,14] The Saudi health authorities recommend vaccination of all pilgrims at the age of 65 years and of those who are at high risk. Despite this recommendation, data show that a vaccination program is not widely implemented; for example, in the 2003 season, the reported influenza vaccination rate was 4.7% among a group of 500 pilgrims.[4] Similarly, vaccination coverage among health-care workers working during Hajj was notably low (5.9%).[15] Clearly, these vaccination rates must be improved by implementing strategies that include education of health-care providers and by making vaccination a prerequisite for acquiring a Hajj visa. At present, the Saudi health authorities do not recommend universal influenza vaccination; it is desirable that all pilgrims, including those at low risk, should receive the influenza vaccination.

Anti-viral chemoprophylaxis has been used in annual influenza epidemics as an adjunct to influenza vaccine. Only 2 drugs, both neuraminidase inhibitors, are currently recommended for preventing or treating influenza: Zanamivir (Relenza) and oseltamir (Tamiflu).[16] Patients should be started on anti-viral medications within 48 hours of contracting ARI. However, these drugs are not widely used due to their high cost. The adamantanes amantadine (Symmetral) and rimantadine (Flumandine) are currently not recommended due to the reported resistance of influenza A (H3N2) virus to these drugs.[17] Although the US CDC suggests that surgical face masks do not provide adequate filtering of small respiratory particles, it may be desirable to use them, particularly in semi-closed areas. Encouraging respiratory hygiene measures such as frequent hand washing and the use of alcohol hand rubs and disinfectants are essential measures in preventing cross-infection.[18]

## Lower Respiratory Tract Infection

Diagnosis and treatment of pneumonia in a mass-gathering situation is a medical challenge requiring quick decision making and knowledge of its etiology. In 2003, pneumonia was the leading cause of hospital admissions during Hajj, (accounting for 39%); and the second leading cause of ICU admissions, at 22%,[2,19] with a reported mortality of 17%.[20] The etiological agents of pneumonia in Hajj have not been extensively studied before; accordingly, the causative agents could differ from those of community-acquired pneumonia.[20] Alzeer *et al.* studied 64 selected patients with pneumonia during the Hajj season of 1994 who failed the first line of therapy and required hospital admission. The diagnosis of pneumonia was confirmed in 46 (72%) patients; *Mycobacterium tuberculosis* (13/46, 28%) and gram-negative organisms (12/46, 26%) were the most common causative organisms, accounting for over half of the culture-confirmed cases. *Streptococcus pneumoniae* was identified in 6 (10%) patients, and atypical organisms were identified in only 4 (6%) patients.[20] The radiographic features of pneumonia in these patients were predominantly in the lower lobes, with lack of cavitations. This could be the result of the acuteness of *M. tuberculosis, which was seen primarily in elderly patients,* and the high incidence of gram-negative organisms.[20] The causes of this high rate of *M. tuberculosis* are multiple; significantly, many pilgrims arrive with active disease in active state from countries endemic for tuberculosis, thus promoting TB spread. Data also suggest that the decreased cell-mediated immunity due to overcrowding, exhaustion and undernourishment can result in a high incidence of pneumonia and, in particular, outbreak of a “caseous” form of pulmonary tuberculosis characterized by severe systemic effect and a high mycobacterium load in a manner that simulates pyogenic pneumonia.[21–23] A recent data from Singapore reveals that using QuantiFIERON TB assay, 10 (15%) of the 149 pilgrims who were negative prior to Hajj had a significant rise in immune response to TB 3 months after Hajj.[24] Moreover, the skin reactivity to TB and annual risk of infection were 3 times higher in Saudi cities hosting pilgrims compared to the corresponding national averages.[25] This was attributed to contact with pilgrims from developing countries that have a high prevalence of tuberculosis. In addition, most of the international pilgrims travel by air, often for a long journey. This may prove to be a significant mode of spread of infection, especially with crowded chartered flights with many susceptible individuals on board.[26,27]

These data highlight the possible high rate of *M. tuberculosis* cross-infection among pilgrims returning from Mecca, who may act as a potential reservoir for TB. From a public health perspective, they emphasize the need for a feasible and well-defined strategy to minimize the risk of infection. Such a strategy should include screening pilgrims by chest x-ray before allowing them to enter Saudi Arabia; and minimizing crowding, particularly by reducing the number and concentration of people housed in tents. Given the high rate of tuberculosis infection and its atypical presentation in patients with pneumonia, an early screening for *M. tuberculosis* in sputum and a potential trial of antituberculous chemotherapy might be indicated in case of failure to respond to ordinary antibiotics. In view of the emergence of multidrug-resistant tuberculosis in highly endemic areas, isoniazid prophylaxis may prove to be ineffective. Taking into account the short period of Hajj and the fact that pilgrims are always in motion, establishing an equipped laboratory with up-to-date molecular biology facilities is necessary for the rapid diagnosis and identification of infectious agents, particularly those causing TB. Data obtained would also be valuable for the international WHO program for the control and elimination of TB worldwide. The subspeciation and antimicrobial susceptibility of these subspecies would be extremely useful for suggesting methods of control.

In summary, respiratory tract infection during Hajj continues to exert a burden on pilgrims. Since data is lacking, the Saudi Ministry of Health must establish a registry for these infections. Future studies should focus on the prevention, diagnosis, epidemiology and management of these respiratory diseases in this large heterogeneous population, as Mecca will always be “once-in-a-lifetime destination” for all capable Muslims. The WHO and ministries of health in countries from which pilgrims originate should cooperate with Saudi authorities for exchange of health-related information.
